# Outbreak of *Corynebacterium pseudodiphtheriticum* Infection in Cystic Fibrosis Patients, France

**DOI:** 10.3201/eid1608.100193

**Published:** 2010-08

**Authors:** Fadi Bittar, Carole Cassagne, Emmanuelle Bosdure, Nathalie Stremler, Jean-Christophe Dubus, Jacques Sarles, Martine Reynaud-Gaubert, Didier Raoult, Jean-Marc Rolain

**Affiliations:** Université de la Méditerranée, Marseille, France (F. Bittar, C. Cassagne, D. Raoult, J.-M. Rolain); Hôpital Timone, Marseille (E. Bosdure, N. Stremler, J.-C. Dubus, J. Sarles); Hôpital Sainte-Marguerite, Marseille (M. Reynaud-Gaubert); 1 These authors contributed equally to this article.

**Keywords:** bacteria, Corynebacterium pseudodiphtheriticum, cystic fibrosis, cough, intact cell mass spectrometry, children, respiratory infections, MALDI-TOF, France, research

## Abstract

An increasing body of evidence indicates that nondiphtheria corynebacteria may be responsible for respiratory tract infections. We report an outbreak of *Corynebacterium pseudodiphtheriticum* infection in children with cystic fibrosis (CF). To identify 18 *C. pseudodiphtheriticum* strains isolated from 13 French children with CF, we used molecular methods (partial *rpoB* gene sequencing) and matrix-assisted laser desorption ionization time-of-flight (MALDI-TOF) mass spectrometry. Clinical symptoms were exhibited by 10 children (76.9%), including cough, rhinitis, and lung exacerbations. The results of MALDI-TOF identification matched perfectly with those obtained from molecular identification. Retrospective analysis of sputum specimens by using specific real-time PCR showed that ≈20% of children with CF were colonized with these bacteria, whereas children who did not have CF had negative test results. Our study reemphasizes the conclusion that correctly identifying bacteria at the species level facilitates detection of an outbreak of new or emerging infections in humans.

## Abstract 2

Respiratory tract colonization with these bacteria may be common in this population.

Cystic fibrosis (CF) is an autosomal recessive disease characterized by defective ion channels, resulting in multiorgan dysfunction, most notably affecting the respiratory tract. The alteration in pulmonary environment is associated with increased susceptibility to bacterial infections (*1**,**2*). These bacterial infections and the ensuing inflammation damage the airway epithelium and cause recurrent episodes of acute exacerbations, leading ultimately to respiratory failure. Respiratory infections account for 80%–90% of deaths of patients with CF (*2*). Recent advances in bacterial taxonomy and improved microbial identification methods have led to increasing recognition of the complexity of microbial ecology of the CF lung (*3**–**5*). Thus, infections of the lung in patients with CF are now considered as polymicrobial infections. In addition to well recognized CF pathogens (e.g., *Staphylococcus aureus*, *Pseudomonas aeruginosa*, *Haemophilus influenzae*, and *Burkholderia cepacia* complex) numerous other opportunistic bacteria have been recently reported, such as *Stenotrophomonas maltophila*, *Achromobacter xylosoxydans*, and *Inquilinus limosus* and methicillin-resistant *S. aureus* and mucoid *P. aeruginosa* (*1**,**2**,**6**–**8*).

The first difficulty in studying infections in the lungs of patients with CF is that many bacteria present in the lung cannot be isolated from sputum samples either because of their fastidious growth requirements or because of the presence of other more common CF-related pathogens, including *P. aeruginosa*, *S. aureus*, *H. influenzae,* and *Branhamella catarrhalis*, that might ordinarily overgrow other bacteria in culture. Second, correct identification of bacteria in patients with CF remains challenging because phenotype variation is a common feature during chronic infection of the lung (*4**,**9*). Consequently, the list of bacteria that can be recovered from sputum specimens of patients with CF may be underestimated, and new or emerging bacteria that could be responsible for outbreaks in this population are not easily detected. Correct identification of these bacteria is not easily achieved.

Several studies have reported the use of matrix-assisted laser desorption ionization time-of-flight (MALDI-TOF) mass spectrometry as a powerful tool with good and reproducible results for rapid identification of clinical isolates in the microbiology laboratory (*10*) as well as for identifying nonfermenting gram-negative bacteria in patients with CF (*11**–**13*). This method is simple, rapid, easy to perform, inexpensive, and may ultimately replace routine phenotypic assays (*10*).

We report the clinical and microbiologic features of patients with CF who were infected or colonized by *C. pseudodiphtheriticum.* The index case-patient was a 9-year-old girl with fever and cough; a coryneform bacterium was isolated in pure culture from her sputum. After this first case, several other children with CF were found to be infected by coryneform bacteria; thus, we decided to investigate the possibility of an endemic transmission in this population. Isolated strains were identified by using existing phenotypic and molecular methods (*14*) as well as MALDI-TOF to decipher the relationship between these strains. Finally, a new real-time PCR with TaqMan probe (Applied Biosystems, Courtaboeuf, France) was developed and used in a retrospective analysis to detect these coryneform bacteria in our population with CF.

## Methods

### Sample Collection and Bacteriologic Culture

From August 2005 through June 2008, sputum samples, bronchoalveolar lavage samples, or both, were collected from patients with CF at 2 cystic fibrosis treatment centers (CFTCs), Timone Children’s Hospital (patients <18 years of age; CFTC1) and Ste. Marguerite Hospital in Marseille (patients >18 years of age; CFTC2). Only samples that showed, by direct Gram staining, infrequent epithelial cells (<10 cells/field) and numerous polymorphonuclear cells (>25 cells/field) were further analyzed and processed according to current local guidelines (*4*). A portion of each sample was also frozen at –20°C for further study. Respiratory samples from patients who did not have CF (children admitted to the pediatric healthcare center and adults admitted to CFTC2) were also collected for control analysis. The *Corynebacterium* reference strains used in this study are listed in the Table. This study was approved by our local ethics committee (no. 07–011).

**Table:** Strains used to test the specificity of quantitative PCR and Ct obtained in a study of *Corynebacterium pseudodiphtheriticum* infection in CF patients, France, August 2005–June 2008*

*Corynebacterium* spp.	Reference	Ct
*C. accolens*	CIP104783T	38
*C. afermentans subsp. afermentans*	CIP103499T	–
*C. afermentans subsp. lipophilum*	CIP103500T	–
*C. amycolatum*	CIP103452T	–
*C. coyleae*	CIP104919T	–
*C. diphteriae*	CIP100721T	–
*C. durum*	CIP105490T	39
*C. freneyi*	CIP106767T	–
*C. glucuronolyticum*	CIP104577T	–
*C. imitans*	CIP105130T	–
*C. jeikeium*	Blood culture	–
*C. macginleyi*	CIP104099T	–
*C. minutissimum*	CIP100652T	–
*C. mucifaciens*	CIP105129T	–
*C. propinquum*	CIP103792T	23
*C. pseudodiphtheriticum*	CIP103420T	21
*C. riegelii*	CIP105310T	38
*C. seminal*	CIP104297T	–
*C. singular*	CIP105491T	–
*C. striatum*	CIP81.15T	–
*C. ulcerans*	CIP106504T	–
*C. urealyticum*	CIP103524T	–
*C. xerosis*	CIP100653T	–
*C. aurimucosum*	CCUG 47449T	–
*C. fastidiosum*	CIP103808	–

*CF, cystic fibrosis; Ct, cycle threshold; –, negative.

### Phenotypic Identification

The positive bacilli from respiratory samples, identified by Gram stain, were investigated by metabolic tests, as oxidase and catalase activities, and by the use of Api (RAPID) Coryne Database 2.0 system (bioMérieux, Marcy-l’Etoile, France) (*15*). The antimicrobial drug susceptibility testing was performed by disk diffusion method on Muelle-Hinton agar with 5% sheep blood incubated for 24 h at 37°C.

### Genotypic Identification and Sequence Analysis

Primers used in this study for amplification and sequencing the partial *rpoB* gene as well as PCR methods have been previously described (*16*). Multiple sequence alignment and percentage of similarities for the partial *rpoB* genes between the different species of corynebacteria were done by using the ClustalW program on the EMBL-EBI web server (www.ebi.ac.uk/clustalw). A phylogenic tree was generated by using the neighbor-joining method from MEGA 4.0 software (www.megasoftware.net). Kimura 2-parameter was used as a substitution model to construct the *rpoB* tree. Bootstrap replicates were performed to estimate the reliabilities of the nodes of the phylogenic tree obtained.

### Bacterial Analysis by MALDI-TOF Mass Spectrometry

The strains were plated on Columbia agar with 5% sheep blood (COS) (bioMérieux) and incubated for 24 h at 37°C. One isolated colony from each strain was harvested and deposited on a target plate (Bruker Daltonics, Bremen, Germany) in 3 replicates. Two microliters of matrix solution (saturated α-cyano-4-hydroxycinnamic acid, 50% acetonitrile, 2.5% trifluoroacetic acid) was then added and samples were processed in the MALDI-TOF mass spectrometry (337 nm) (Autoflex, Bruker Daltonics with the flex control software) (*10*). The profiles were compared and analyzed by Biotyper 2.0 (Bruker Daltonics) and finally a dendrogram of mass spectral data was constructed by using the instructor default setting. The Biotyper 2.0 program generates the tree depending on distance-based method and does not provide branch support values.

### Real-time PCR

A new real-time PCR with a TaqMan probe (Applied Biosystems) that targets the *rpoB* gene of *C. pseudodiphtheriticum* has been developed and tested retrospectively in sputum samples that had previously been collected in a 1-year study from January through December 2006. Sputum samples from 4 groups of patients were analyzed: sputum samples from child (group 1) and adult (group 2) CF patients and sputum samples from non-CF children (group 3) and from non-CF adults (group 4). Primers and probe used were as follows: CorynPF (5′-GACGGYGCTTCCAACGAAGA-3′) and CorynPR (5′-CCGACGGAGATCGGGTGC-3′) and probe CorynPr (6FAM-TCTGTTGGCTAACTCCCGYCCAAA-TAMRA). Specificity of these primers and probe was verified in silico by using the BLAST program (www.ncbi.nlm.nih.gov/BLAST) as well as by using corynebacteria reference strains (Table). Sensitivity was assessed by using tenfold serial dilutions of a 0.5 MacFarland inoculum.

## Results

### Patients and Samples

Overall, 229 patients with CF were monitored from August 2005 through June 2008 in the 2 CFTCs in Marseille (118 children and 111 adults). During this period, 18 corynebacteria were isolated from respiratory samples of 13 children with CF (11.0%) but none from adults with CF (p<0.001). Details for the 13 patients are given in the Table A1. The mean age was 4.3 (0.3–16) years, and the sex ratio (M:F) was 0.6. Isolation of *C. pseudodiphtheriticum* was associated with clinical symptoms in 10 patients (76.9%), including cough, rhinitis, asthma crisis, and lung exacerbations (Table A1). The culture of *C. pseudodiphtheriticum* from respiratory samples was pure in 6 cases (in 2 cases, patients had clinical symptoms). For 4 patients, a *Corynebacterium* isolate was obtained on >1 occasion (Table A1). Six patients were treated, including 3 with β-lactams only, 1 with a combination of a β-lactam and cotrimoxazole, 1 with cotrimoxazole alone, and 1 with cotrimoxazole alone initially and then amoxicillin because no improvement was noticed and the isolate was resistant to cotrimoxazole.

### Phenotypic and Molecular Identification of the Isolates

**Figure 1:**
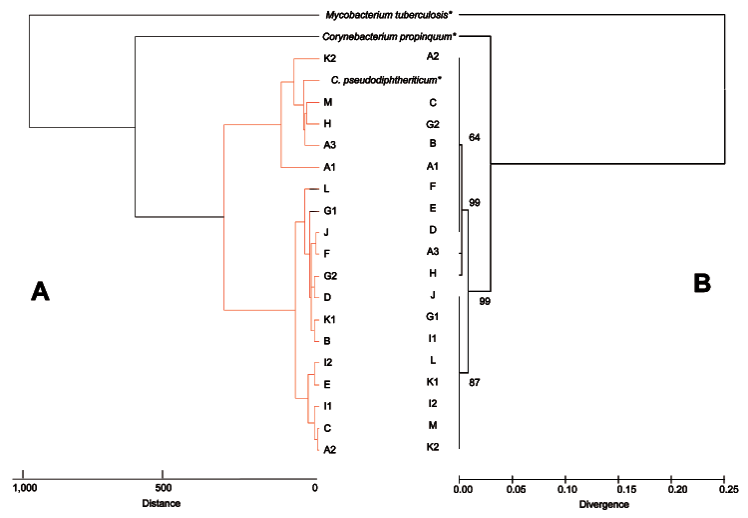
Phylogenetic tree showing the position of *Corynebacterium* spp. isolated in patients with cystic fibrosis based on comparisons of the mass spectra obtained with matrix-assisted laser desorption ionization time-of-flight (MALDI-TOF) mass spectrometry (A) and of sequences of the partial RNA polymerase β-subunit gene *rpoB* (B). For MALDI-TOF, a tree was constructed with Biotyper 2.0 software (Bruker Daltonics, Bremen, Germany) using Euclidean distance. The *rpoB* tree was constructed by the neighbor-joining method and a maximum likelihood-based distance algorithm and numbers on branches indicate the bootstrap values derived from 500 replications. *Reference strains (*Mycobacterium tuberculosis* H37Rv, *C. propinquum* CIP103792T, *C. pseudodiphtheriticum* CIP103420T).

All corynebacteria were isolated from Columbia agar with 5% sheep blood. Colonies were white and nonhemolytic. They were all catalase positive and oxidase negative. The use of the ApiCoryne 2.0 system yielded identification of 16 *C. pseudodiphtheriticum* and 1 *C. propinquum*, with a confidence level 83%–99% (Table A1). The remaining isolate was poorly identified as *Brevibacter* sp. with an uninterpretable pattern (Table A1). All isolates were susceptible to β-lactams, vancomycin, rifampin, gentamicin, and doxycycline, whereas there was heterogeneity of susceptibility for erythromycin and cotrimoxazole (Table A1). The partial *rpoB* gene sequencing provided an accurate identification for 18 isolates (A1 to M) with similarity >97% compared with reference strains (Table A1). The results of the MALDI-TOF identification matched perfectly with the partial *rpoB* sequencing identification for all the isolates; mean score values ranged from 1.8–2.5 (Table A1). Figure 1 presents 2 trees built by using MALDI-TOF mass spectrometry (Figure 1, panel A) and by using partial *rpoB* gene sequences (Figure 1, panel B). Although comparison between these 2 trees was impossible because of the different methods used (i.e., Euclidean distance method for MALDI-TOF dendrogram and neighbor-joining method for phylogenetic tree), the 2 trees gave a similar clustering of the isolates (Figure 1).

### Real-time PCR

**Figure 2:**
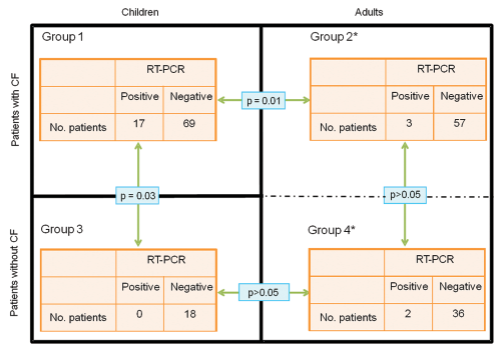
Results of real-time quantitative PCR specific for the *rpoB* gene for the detection of *Corynebacterium pseudodiphtheriticum* in sputum samples for the 4 groups of patients from 3 separate healthcare centers in Marseille, France, from January through December 2006. Black lines separate the different healthcare centers. Group 1, cystic fibrosis (CF) treatment center (CFTC) 1, with children with CF; group 2, CFTC2, with adults with CF; group 3, center with children without CF; group 4, patients without CF. *Patients from groups 2 and 4 hospitalized in the same healthcare center (CFTC2).

Sensitivity of our new real-time PCR ranged from 5 CFU/mL to 10 CFU/mL; specificity for *C. pseudodiphtheriticum* was verified by testing *Corynebacterium* spp. reference strain cultures with *C. propinquum*, the most closely related species, which was also amplified. A low level of cross-amplification was also observed in 3 other species with cycle thresholds (Ct) >38 cycles, including *C. accolens*, *C. durum*, and *C. riegelii* (Table); this finding could not be considered clinically relevant. To estimate the prevalence of this bacterium in our CF population, we used the real-time PCR to test, retrospectively and blindly, all respiratory samples available from January through December 2006 from 146 patients with CF (n = 356 sputum samples; 86 children, group 1; and 60 adults, group 2) and from 56 patients without CF (n = 67 sputum samples; 18 children, group 3; and 38 adults, group 4). We found 24 PCR-positive sputum specimens (Ct <37) in 17 children (19.8%) and 3 adults (5%) in patients with CF (Figure 2). Among these 24 PCR-positive samples only 2 were culture positive (sample A1, Table A1) (p<0.0001). Thus, 16 additional children and 3 adult patients with CF were eventually colonized with this bacterium. For the control group, although all samples were culture negative, we found 3 PCR-positive samples in 2 adult patients, who were followed up in CFTC2 for lung transplantation, and none from children (Figure 2). The 2 PCR-positive lung-transplant patients were hospitalized in the adult CFTC with 2 adult patients with CF during the same period. For these 2 patients, cultures of sputum samples were polymicrobial, and findings were interpreted as normal flora. Finally, the number of PCR-positive children with CF was significantly higher than the number of either children without CF or adults with CF (p = 0.03 and p = 0.01, respectively; Figure 2). Conversely, this difference was not significant between adult patients with CF and adult patients without CF (Figure 2). Notably, the 18 children who did not have CF were seen by clinicians in the same hospital, but not in the same healthcare center, and were not in contact with CF children. Thus, the only risk factor found for being infected or colonized with *C. pseudodiphtheriticum* was to be monitored at the CF center.

## Discussion

We report the isolation of *C. pseudodiphtheriticum* in children with CF who had respiratory disease, mainly cough and rhinitis. As reemphasized in our study, this group of organisms is poorly identified by current phenotypic methods that lack specificity and result in ambiguous or even erroneous identification. These bacteria are usually considered as part of the natural flora of the respiratory tract, skin, and mucous membranes (*17*) and are not reported to clinicians. Moreover, culture of bacteria from sputum samples of patients with CF is known to lack sensitivity, either because of the fastidious nature of several organisms or because of overgrowth by common bacteria such as mucoid *P. aeruginosa* (*4**,**5*). For these reasons, an outbreak in a specific population of patients such as patients with CF may easily go unnoticed.

Our study shows that a correct identification of bacteria remains critical for detecting such a possibility and that surveillance of the circulation of bacteria within patients with CF should be addressed in the future so new or emerging pathogens can be detected. For this purpose, we eventually identified the isolates by using PCR amplification and sequencing of the *rpoB* gene (currently the standard method) (*16*) and compared the findings to those obtained with the MALDI-TOF mass spectrometry method. Interestingly, the bacteria identified were exactly the same with both methods. This result suggests MALDI-TOF may represent a rapid inexpensive alternative assay for identification of these bacteria at the species level as recently reported for routine identification of bacteria (*10*). Moreover, both methods were highly discriminatory and allowed us to demonstrate that patients were infected or colonized by *C. pseudodiphtheriticum*. The dendogram obtained with the MALDI-TOF technique for identification of *C. pseudodiphtheriticum* was in general agreement with that of a partial *rpoB* gene sequencing phylogenetic tree, but identification of the strains at the species level could be obtained within minutes. Further studies to evaluate the typing power of MALDI-TOF mass spectrometry to discriminate bacterial strains below the species level should be done. In addition, the correct identification of the bacteria was the critical step in designing a new tool, i.e., a specific real-time quantitative PCR, to investigate the presence of an outbreak in the CF population.

The importance of positive cultures for nondiphtheria corynebacteria obtained from clinical samples of patients with signs and symptoms should not be overlooked (*18*). Although nondiphtheria corynebacteria were historically considered as contaminants without clinical significance, an increasing body of evidence shows their pathogenicity, especially as a cause of nosocomial infection in hospitalized and immunocompromised patients (*19*). Among coryneform bacteria, *C. pseudodiphtheriticum* and *C. striatum* have been well documented as pathogens of the respiratory tract, leading to nosocomial and community-acquired pneumonia (*18**,**20**,**21*) as well as bronchitis, tracheitis, lung exacerbation, chronic obstructive lung disease, and lung abscesses (*22**–**29*).

In our study, we were initially surprised to isolate these bacteria in pure culture from sputa of patients with CF. About 70% of the patients had pulmonary symptoms, especially cough, and 6 (46.2%) case-patients required antimicrobial drug treatment. It is noteworthy that these clinical symptoms may be due either to coryneform toxins or to other respiratory pathogens, including viruses that were not investigated in this study. Surprisingly, 4 of 13 children had >1 isolate during the study period, which suggests that patients with CF become chronically colonized with *C. pseudodiphtheriticum*. Because of the difficulty in isolating these bacteria in respiratory samples, except for those that are in pure culture, their prevalence within the CF population may well be underestimated. This hypothesis was supported by the retrospective detection of DNA in additional sputum specimens from children with CF whose culture results were negative (≈20% of positive children) and adult patients by using our specific real-time PCR. We found that *C. pseudodiphtheriticum* was significantly associated with children with CF, which suggests transmission between patients with CF may have occurred in the CF healthcare center because none of the children without CF who were seen in a separate healthcare center (a different floor in the hospital) were PCR positive.

Patient-to-patient transmission could not be excluded and should be further investigated because 2 adult patients without CF who had PCR-positive specimens were detected in the same adult CFTC where they likely had contact with 2 PCR-positive CF adult patients. Such transmission has been recently demonstrated for *C. striatum* as a cause of nosocomial outbreak and respiratory colonization in patients with chronic obstructive pulmonary disease (*30*). Similarly, an outbreak of clonal multidrug-resistant strains of *C. striatum* as an emerging agent of pulmonary disease has been recently reported in Italy (*21*). Further epidemiologic studies are warranted to define the role of *C. pseudodiphtheriticum* transmission in the course of CF disease in other CF centers. Finally we believe that the implementation of isolation or segregation measures should be the rule in CF centers to reduce the risk of transmission of pathogens.

In conclusion, corynebacteria may colonize the respiratory tract of CF patients. Although the clinical importance of *C. pseudodiphtheriticum* in the complex setting of CF patients is less clear, we believe that this bacterium should be added in the list of new or emerging pathogens in these patients. Further clinical studies are needed to establish whether corynebacteria may contribute to the pathology of lung disease in CF patients.

## Appendix

**Table A1:** Clinical data of patients with cystic fibrosis, with phenotypic and genotypic identification of *Corynebacterium* isolates, France, 2006*

Patient	Age/sex	Isolate	Symptoms	Treatment	Sample	Other isolated pathogens	Identification										Ery	SXT
							ApiCoryne				*rpo*B PCR				MALDI-TOF MS			
							Result	%	Code		Result	%	Accession no.		Result	Score value†		
A	4 mo/F	A1	Cough	Unknown	BAL	SA, fungi	CP	96.8	3001004		CP	99	AY492232		CP	1,815	R	S
		A2	None	Unknown	BAL	None	CP	96.8	3001004		CP	99	AY492232		CP	2,492	R	S
		A3	Unknown	Unknown	BAL	None	CP	99	7001004		CP	99	AY492232		CP	1,950	R	I
B	35 mo/F	B	Rhinitis	None	NPA	HI	CP	96.8	3001004		CP	97	AY492232		CP	2,051	R	S
C	5 y/M	C	None	None	SP	SA	CP	84.6	3003004		CP	100	AY492232		CP	2,477	S	S
D	10 y/F	D	None	None	SP	CAL	CP	94.9	3101004		CP	100	AY492232		CP	2,272	I	S
E	13 mo	E	Unknown	Unknown	BAL	None	Br	UIP	5101764		CP	100	AY492232		CP	2,353	S	S
F	2 y/M	F	Cough	AMX and SXT	BAL	SA, fungi	CP	94.9	3101004		CP	99	AY492232		CP	2,416	S	I
G	15 mo/F	G1	Cough	AUG	NPA	SA	CP	97.9	5101007		CP	97	AY492232		CP	2,246	R	S
		G2	None	None	NPA	None	CPQ	88	3000004		CP	100	AY492232		CP	2,069	R	S
H	35 mo/F	H	Rhinitis, cough	AMX	NPA	SA	CP	92.3	1021004		CP	99	AY492232		CP	1,914	R	S
I	2 mo/F	I1	Cough	None	NPA	EC	CP	1.1	2001004		CP	98	AY492232		CP	2,444	R	S
		I2	Cough	None	NPA	EC	CP	95	3101004		CP	98	AY492232		CP	2,406	S	S
J	9 y/F	J	Cough	SXT then AMX	SP	None	CP	94.9	3101004		CP	98	AY492232		CP	2,461	S	S
K	21 mo/M	K1	Rhinitis, cough	AUG	NPA	BC	CP	97.9	5101004		CP	98	AY492232		CP	2,386	R	S
		K2	Rhinitis	AUG	NPA	HI	CP	94.9	3101004		CP	98	AY492232		CP	2,044	R	S
L	3 y/M	L	Cough	SXT	NPA	BC	CP	94.9	3101004		CP	98	AY492232		CP	2,098	R	S
M	16 y/F	M	Rhinitis	None	SP	None	CP	96.8	3001004		CP	98	AY492232		CP	1,787	S	R

*MALDI-TOF MS, matrix-assisted laser desorption/ionization time-of-flight mass spectrometry; Ery, erythromycin; SXT, cotrimoxazole; BAL, bronchoalveolar fluid; SA, *Staphylococcus aureus*; CP, *Corynebacterium pseudodiphtheriticum*; R, resistant; S, sensitive; I, intermediary; NPA, nasopharyngeal aspirate; SP, sputum sample; HI, *Haemophilus influenzae*; CAL, *Candida albicans*; Br, *Brevibacterium sp*; UIP, uninterpretable pattern; AMX, amoxicillin; AUG, amoxicillin and clavulanic acid; CPQ, *Corynebacterium propinquum*; EC, *Escherichia coli*; BC, *Branhamella catarrhalis*. †Score value represents the mean of 3 different analyses.

## References

[1] Saiman L, Siegel J. Infection control in cystic fibrosis. Clin Microbiol Rev. 2004;17:57–71. 10.1128/CMR.17.1.57-71.2004 14726455 PMC321464

[2] Lyczak JB, Cannon CL, Pier GB. Lung infections associated with cystic fibrosis. Clin Microbiol Rev. 2002;15:194–222. 10.1128/CMR.15.2.194-222.2002 11932230 PMC118069

[3] Harrison F. Microbial ecology of the cystic fibrosis lung. Microbiology. 2007;153:917–23. 10.1099/mic.0.2006/004077-0 17379702

[4] Bittar F, Richet H, Dubus JC, Reynaud-Gaubert M, Stremler N, Sarles J, Molecular detection of multiple emerging pathogens in sputa from cystic fibrosis patients. PLoS ONE. 2008;3:e2908. 10.1371/journal.pone.0002908 18682840 PMC2483419

[5] Harris JK, De Groote MA, Sagel SD, Zemanick ET, Kapsner R, Penvari C, Molecular identification of bacteria in bronchoalveolar lavage fluid from children with cystic fibrosis. Proc Natl Acad Sci U S A. 2007;104:20529–33. 10.1073/pnas.0709804104 18077362 PMC2154465

[6] Lambiase A, Raia V, Del PM, Sepe A, Carnovale V, Rossano F. Microbiology of airway disease in a cohort of patients with cystic fibrosis. BMC Infect Dis. 2006;6:4. 10.1186/1471-2334-6-4 16405721 PMC1351191

[7] Bittar F, Leydier A, Bosdure E, Toro A, Boniface S, Stremler N, *Inquilinus limosus* and cystic fibrosis. Emerg Infect Dis. 2008;14:993–5. 10.3201/eid1406.071355 18507928 PMC2600277

[8] Rolain JM, Francois P, Hernandez D, Bittar F, Richet H, Fournous G, Genomic analysis of an emerging multiresistant *Staphylococcus aureus* strain rapidly spreading in cystic fibrosis patients revealed the presence of an antibiotic inducible bacteriophage. Biol Direct. 2009;4:1. 10.1186/1745-6150-4-1 19144117 PMC2629466

[9] Kiska DL, Kerr A, Jones MC, Caracciolo JA, Eskridge B, Jordan M, Accuracy of four commercial systems for identification of *Burkholderia cepacia* and other gram-negative nonfermenting bacilli recovered from patients with cystic fibrosis. J Clin Microbiol. 1996;34:886–91. 8815102 10.1128/jcm.34.4.886-891.1996 PMC228911

[10] Seng P, Drancourt M, Gouriet F, La Scola B, Fournier PE, Rolain JM, On-going revolution in bacteriology: routine identification by matrix-assisted laser desorption ionization time-of-flight mass spectrometry. Clin Infect Dis. 2009;49:543–51. 10.1086/600885 19583519

[11] Degand N, Carbonnelle E, Dauphin B, Beretti JL, Le BM, Sermet-Gaudelus I, Matrix-assisted laser desorption ionization-time of flight mass spectrometry for identification of nonfermenting gram-negative bacilli isolated from cystic fibrosis patients. J Clin Microbiol. 2008;46:3361–7. 10.1128/JCM.00569-08 18685005 PMC2566097

[12] Vanlaere E, Sergeant K, Dawyndt P, Kallow W, Erhard M, Sutton H, Matrix-assisted laser desorption ionisation-time-of-flight mass spectrometry of intact cells allows rapid identification of *Burkholderia cepacia* complex. J Microbiol Methods. 2008;75:279–86. 10.1016/j.mimet.2008.06.016 18627778

[13] Minan A, Bosch A, Lasch P, Stammler M, Serra DO, Degrossi J, Rapid identification of *Burkholderia cepacia* complex species including strains of the novel Taxon K, recovered from cystic fibrosis patients by intact cell MALDI-ToF mass spectrometry. Analyst (Lond). 2009;134:1138–48. 10.1039/b822669e 19475140

[14] Khamis A, Raoult D, La SB. Comparison between *rpoB* and 16S rRNA gene sequencing for molecular identification of 168 clinical isolates of *Corynebacterium.* J Clin Microbiol. 2005;43:1934–6. 10.1128/JCM.43.4.1934-1936.2005 15815024 PMC1081344

[15] Funke G, Renaud FN, Freney J, Riegel P. Multicenter evaluation of the updated and extended API (RAPID) Coryne database 2.0. J Clin Microbiol. 1997;35:3122–6. 9399506 10.1128/jcm.35.12.3122-3126.1997 PMC230134

[16] Khamis A, Raoult D, La SB. *rpoB* gene sequencing for identification of *Corynebacterium* species. J Clin Microbiol. 2004;42:3925–31. 10.1128/JCM.42.9.3925-3931.2004 15364970 PMC516356

[17] von GA. Punter-Streit V, Riegel P, Funke G. Coryneform bacteria in throat cultures of healthy individuals. J Clin Microbiol. 1998;36:2087–8. 9650969 10.1128/jcm.36.7.2087-2088.1998 PMC104985

[18] Camello TC, Souza MC, Martins CA, Damasco PV, Marques EA, Pimenta FP, *Corynebacterium pseudodiphtheriticum* isolated from relevant clinical sites of infection: a human pathogen overlooked in emerging countries. Lett Appl Microbiol. 2009;48:458–64. 10.1111/j.1472-765X.2009.02553.x 19228291

[19] Bernard KA, Munro C, Wiebe D, Ongsansoy E. Characteristics of rare or recently described corynebacterium species recovered from human clinical material in Canada. J Clin Microbiol. 2002;40:4375–81. 10.1128/JCM.40.11.4375-4381.2002 12409436 PMC139690

[20] Manzella JP, Kellogg JA, Parsey KS. *Corynebacterium pseudodiphtheriticum*: a respiratory tract pathogen in adults. Clin Infect Dis. 1995;20:37–40. 7727667 10.1093/clinids/20.1.37

[21] Campanile F, Carretto E, Barbarini D, Grigis A, Falcone M, Goglio A, Clonal multidrug-resistant *Corynebacterium striatum* strains, Italy. Emerg Infect Dis. 2009;15:75–8. 10.3201/eid1501.080804 19116057 PMC2660704

[22] Freeman JD, Smith HJ, Haines HG, Hellyar AG. Seven patients with respiratory infections due to *Corynebacterium pseudodiphtheriticum.* Pathology. 1994;26:311–4. 10.1080/00313029400169721 7991290

[23] Colt HG, Morris JF, Marston BJ, Sewell DL. Necrotizing tracheitis caused by *Corynebacterium pseudodiphtheriticum*: unique case and review. Rev Infect Dis. 1991;13:73–6. 2017636 10.1093/clinids/13.1.73

[24] Miller RA, Rompalo A, Coyle MB. *Corynebacterium pseudodiphtheriticum* pneumonia in an immunologically intact host. Diagn Microbiol Infect Dis. 1986;4:165–71. 10.1016/0732-8893(86)90152-5 3956138

[25] Chiner E, Arriero JM, Signes-Costa J, Marco J, Corral J, Gomez-Esparrago A, *Corynebacterium pseudodiphtheriticum* pneumonia in an immunocompetent patient. Monaldi Arch Chest Dis. 1999;54:325–7. 10546474

[26] Martaresche C, Fournier PE, Jacomo V, Gainnier M, Boussuge A, Drancourt M. A case of *Corynebacterium pseudodiphtheriticum* nosocomial pneumonia. Emerg Infect Dis. 1999;5:722–3. 10.3201/eid0505.990517 10610204 PMC2627726

[27] Craig TJ, Maguire FE, Wallace MR. Tracheobronchitis due to *Corynebacterium pseudodiphtheriticum.* South Med J. 1991;84:504–6. 10.1097/00007611-199104000-00026 2014440

[28] Cimolai N, Rogers P, Seear M. *Corynebacterium pseudodiphtheriticum* pneumonitis in a leukaemic child. Thorax. 1992;47:838–9. 10.1136/thx.47.10.838 1481190 PMC464074

[29] Gutierrez-Rodero F, Ortiz de la Tabla V, Martinez C, Masia MM, Mora A, Escolano C, *Corynebacterium pseudodiphtheriticum*: an easily missed respiratory pathogen in HIV-infected patients. Diagn Microbiol Infect Dis. 1999;33:209–16. 10.1016/S0732-8893(98)00163-1 10212746

[30] Renom F, Garau M, Rubi M, Ramis F, Galmes A, Soriano JB. Nosocomial outbreak of *Corynebacterium striatum* infection in patients with chronic obstructive pulmonary disease. J Clin Microbiol. 2007;45:2064–7. 10.1128/JCM.00152-07 17409213 PMC1933039

